# Raw Acceleration from Wrist- and Hip-Worn Accelerometers Corresponds with Mechanical Loading in Children and Adolescents

**DOI:** 10.3390/s23156943

**Published:** 2023-08-04

**Authors:** Gemma Brailey, Brad Metcalf, Lisa Price, Sean Cumming, Victoria Stiles

**Affiliations:** 1 Department of Public Health and Sports Sciences, Faculty of Health and Life Sciences, University of Exeter, St. Luke’s Campus, Exeter EX1 2LU, UK; b.metcalf@exeter.ac.uk (B.M.); l.r.s.price@exeter.ac.uk (L.P.); v.h.stiles@exeter.ac.uk (V.S.); 2 Department for Health, University of Bath, Bath BA2 7AY, UK; s.cumming@bath.ac.uk

**Keywords:** accelerometers, bone, impact loading, ground reaction force, physical activity, children and adolescents

## Abstract

The purpose of this study was to investigate associations between peak magnitudes of raw acceleration (g) from wrist- and hip-worn accelerometers and ground reaction force (GRF) variables in a large sample of children and adolescents. A total of 269 participants (127 boys, 142 girls; age: 12.3 ± 2.0 yr) performed walking, running, jumping (<5 cm; >5 cm) and single-leg hopping on a force plate. A GENEActiv accelerometer was worn on the left wrist, and an Actigraph GT3X+ was worn on the right wrist and hip throughout. Mixed-effects linear regression was used to assess the relationships between peak magnitudes of raw acceleration and loading. Raw acceleration from both wrist and hip-worn accelerometers was strongly and significantly associated with loading (all *p*’s < 0.05). Body mass and maturity status (pre/post-PHV) were also significantly associated with loading, whereas age, sex and height were not identified as significant predictors. The final models for the GENEActiv wrist, Actigraph wrist and Actigraph hip explained 81.1%, 81.9% and 79.9% of the variation in loading, respectively. This study demonstrates that wrist- and hip-worn accelerometers that output raw acceleration are appropriate for use to monitor the loading exerted on the skeleton and are able to detect short bursts of high-intensity activity that are pertinent to bone health.

## 1. Introduction

Mechanical loading from physical activity (PA) is one of the most potent modifiable factors that can optimise bone health during growth and reduce the risk of osteoporosis later in life [1]. Assessment of mechanical loading has typically been confined to the assessment of ground reaction forces (GRF) and force loading rates in a laboratory setting. To more closely understand the intricacies of how bone responds to PA during growth and maturation, it is important to be able to accurately assess mechanical loading (GRF) during habitual PA in free-living situations [2]. The use of accelerometers to measure free-living PA has become ubiquitous in research [3,4,5,6]. However, these wearable devices are most frequently used to assess energy expenditure in relation to cardiometabolic health outcomes, and their ability to assess activity related mechanical forces that are more relevant to bone health has been less explored [7].

A handful of previous studies have demonstrated the potential for accelerometers to measure the GRFs incurred during PA in children and adolescents [7,8,9]. However, the use of accelerometers that output proprietary, count-based data limits the comparability and applicability of study findings [6], and, most notably, aggregating the output into epochs results in over-smoothing of the data [10]. As a consequence, dynamic, high-impact activities such as jumping, that are pertinent to bone health [11,12,13] and generate large peak forces at high rates (typically lasting less than 1-s in duration [14]), will go undetected using these methods. Recent technological developments have led to the widespread availability of the raw acceleration signal, which overcomes these limitations and has the resolution necessary to identify impact peaks in the data, making it more reflective of the GRFs experienced in everyday life [15].

In adults, raw acceleration from both wrist- [15,16] and hip-worn [15,16,17,18] devices has been shown to provide a suitable measure of the impact peaks and mechanical loading incurred during PA. Similar findings have also been reported for hip-worn accelerometers in children [14,19]. However, raw acceleration from a wrist-worn device has not been considered in this population. Wrist-worn accelerometers are more acceptable for children and adolescents to wear [20,21] and are increasingly used in large-scale cohort studies including NHANES 2011–2014, Australia’s Child Health CheckPoint [22] and the Pelotas Birth Cohort [23] due to the increased adherence to monitor wear, and more representative estimates of activity behaviour are therefore obtained [20,24,25]. Existing studies in children and adolescents are also limited by small sample sizes (*n* = 13 in [19] and 14 in [14]) and broad age range (5–16 years in [19] and 6–21 years in [14]) of participants. During growth and maturation, a number of physiological, biomechanical and structural changes occur, including (but not limited to) changes in stature, leg length and stride frequency, which influence movement economy and accelerometer output, and changes in landing force and the rate of force application, which influence the loading characteristics obtained during movement [26,27,28,29,30]. It is, therefore, unclear whether raw acceleration magnitudes (from both hip- and wrist-worn accelerometers) across a range of activity intensities reflect the pattern of force and loading rate experienced in children and adolescents as they mature. Understanding this is crucial for the future development, application and interpretation of methods that detect bone-specific activity from hip- and wrist-worn accelerometers in this population.

In light of the preceding discussion, this study aims to investigate the associations between peak magnitudes of raw acceleration from hip- and wrist-worn accelerometers and ground reaction force variables in a large sample of children and adolescents aged 8–16 years and determine whether factors pertaining to growth and maturation influence the associations observed. This will help to determine whether raw acceleration from monitors worn at both wear locations can be used to measure PA in relation to loading in the future and provide researchers with important information regarding the factors that may need to be considered in order to develop methods that are able to do this effectively.

## 2. Materials and Methods

### 2.1. Participants

A total of 282 children were recruited from local schools in and around Exeter, UK. Study information packs containing an information sheet, parental consent form, child assent form and medical screening questionnaire were sent home to all pupils in classes whose physical education lessons coincided with the pre-arranged dates for data collection. Potential participants had a period of two weeks in which they could return their forms to confirm participation in the study. Written informed consent and assent was obtained from parents/guardians and children, respectively. Prior to the collection of data, ethics approval for the study was granted by the University of Exeter Sport and Health Sciences Ethics Committee (ref: 170315/B/01 and 171206/B/10).

### 2.2. Anthropometric Measures

The data collection was conducted in the main school hall/sports hall and took place in two waves, with children aged 9–11 completing the study between June and July of 2017 (Wave 1) and children aged 12–16 from March to July of 2018 (Wave 2). Anthropometric measures were collected for body mass, stature and sitting height according to the methods outlined by Ross et al. [31]. Both stature and sitting height were measured to the nearest 0.1 cm using a portable stadiometer (Leicester Height Measure; Seca, Birmingham, UK), and body mass was measured to the nearest 0.1 kg using an electronic scale (Seca 7802317004, Birmingham, UK). Two measurements were recorded for each, and the mean values were reported. If the values differed by more than 0.4 cm for stature and sitting height and 0.4 kg for weight, a third measure was taken, and the median value was reported. Body mass index (BMI) was then calculated from these measurements using the formula body mass/height^2^ (kg/m^2^). Leg length was calculated by subtracting the subject’s sitting height from their stature. Biological maturity was assessed as maturity offset in years from the estimated age of peak height velocity (PHV) using the sex-specific Mirwald equations [32]. Maturity offset was calculated by subtracting the predicted age of PHV from the participant’s current chronological age.

### 2.3. Procedure

After a sufficient warm-up and familiarisation with each activity, participants performed walking, running, low jumps, higher jumps and hopping on a portable force platform in a randomised order. Children in Wave 1 of data collection completed the activities on a portable AccuSway PLUS force platform (Advanced Mechanical Technology Inc., Watertown, MA, USA; 50.2 × 50.2 × 4.5 cm), which uses Hall Effect sensors to measure ground reaction forces. The force plate was connected to a laptop via a USB 2.0 connection and sampled at a frequency of 200 Hz using the AMTI NetForce software (version 3.5.3). Children in Wave 2 completed the activities on a portable AccuPower force platform (Advanced Mechanical Technology Inc., Watertown, MA, USA; 102 × 76.2 × 12.5 cm). This platform also uses Hall Effect sensors and was connected to a laptop via a USB 2.0 connection and sampled ground reaction force data at a frequency of 1000 Hz using the Accupower software (version 2.0). For each force plate, a runway of 10 m in length topped with a 10 mm depth of EVA foam was constructed so that it was flush with the force plate that was positioned at the midway point. Participants wore sports shoes and performed the walking and running activities in shuttles along this runway for 60 s. A metronome set to 120 beats per minute was used for walking and 190 beats per minute for running. A member of the research team also performed the activities alongside the children to ensure that they stayed in time and made correct contact with the plate without altering their natural gait.

Low jumps (approximately 2–5 cm; 120 beats per minute), higher jumps (>5 cm; 90 beats per minute) and single-leg hopping (2–5 cm 130 beats per minute) were performed continuously on the force plate for 10 s. Between each activity, the force plate was adjusted so that the mass was zeroed before the subject stepped onto the plate. Participants stepped onto the plate when instructed to by the researcher and performed the activities alongside a member of the research team in time with the metronome beat to ensure consistency in jump height. Children were instructed to jump with a slight knee bend, maintain a straight posture and land with their knees slightly bent. There were no restrictions placed on arm movement.

### 2.4. Accelerometry

During testing children wore a triaxial GENEActiv (dynamic range ± 8 g, Activinsights, Kimbolton, Cambridgeshire, UK) accelerometer on the left wrist and an Actigraph GT3X+ (dynamic range ± 6 g, Actigraph, Pensacola, FL 32502, USA) accelerometer on the right wrist. An Actigraph GT3X+ was also worn on the right hip, secured with an elasticated belt. GENEActiv software (version 2.2) and Actilife Software (version 6.0) were used to initialise the accelerometers to collect raw acceleration data at a frequency of 100 Hz (in accordance with previous studies [1,16,19]) and to upload data.

### 2.5. Data Analysis

The magnitude of strain (resulting from gravitational and muscular forces) is an important factor in determining the adaptive response in bone [33]. The external ground reaction force has been shown to be proportional to the magnitude of strain exerted on the skeleton [34] and is therefore a suitable proxy measure of strain magnitude [15,16]. As a high strain magnitude alone may not be sufficient to activate bone cells and a high strain rate is required to stimulate new bone formation [35], the rate of application of external ground reaction forces (loading rate) is also used as a proxy measure of strain rate [15,16]. Output variables from the force plate were therefore the peak vertical ground reaction force (PVF) normalised to bodyweight (BW) calculated by the equation

(1) $$\frac{output\;force\;(N)}{mass\;\left(kg\right)\times 9.81\;{m.s}^{-2}}$$

and the average loading rate (ALR; BW/s) calculated by the equation

(2) $$\frac{peak\;vertical\;force\;(BW)}{time\;of\;peak\;force\;(s)-start\;time\;of\;ground\;contact\;(s)}$$

For the walking and running activities, steps were viewed individually in Excel to ensure correct contact had been made with the force plate, and any incomplete steps were excluded. Ground contact was defined as the period of time in which the force went above 10 N up until it then went below this again. Ground reaction force variables were extracted for at least 4 steps for both walking and running activities. For the low jumps, higher jumps and hopping activities, the force-time histories containing the multiple jumps over the 10 s sampling interval were inspected in Excel, and GRF variables were calculated for a mean of 8 jumps and hops.

Accelerometer files were downloaded and saved in .csv format and exported into Excel for data processing. Resultant acceleration was calculated using the Euclidean norm minus 1 (ENMO) approach for the wrist worn GENEActiv and Actigraph GT3X+ accelerometers using the following equation [36]:

(3) $$\sqrt{\left(x^{2}\right)+\left(y^{2}\right)+(z^{2})}-1$$

For the hip worn GT3X+ accelerometer, only the vertical acceleration was extracted as most of the loading through the body is in line with the vertical vector [15]. The raw resultant and vertical acceleration data was extracted into excel separately for each participant and activity based on the timestamp that was recorded at the start and end of each activity during the data collection. Acceleration-time histories were created for the individual activities and then inspected to identify the peaks in acceleration (maximum values per step/jump that were consistent for each individual within an activity) for all included activities. The peak resultant and vertical magnitudes of raw acceleration were extracted manually for 8–10 steps, jumps and hops, and a mean value was calculated and reported. Accelerometer output was in gravity-based acceleration units (g), where 1 g is equivalent to 9.81 m.s^−2^.

### 2.6. Statistical Analysis

Sample characteristics and descriptive data are presented as mean and standard deviation for continuous variables and as percentages for categorical variables. Independent samples t-tests were used to test for differences between boys and girls for these variables. Mixed-effects linear regression was used to assess the relationship between peak magnitudes of raw acceleration from each accelerometer (GENEActiv wrist, Actigraph wrist and Actigraph hip) and force to account for the fact that participants had performed repeated assessments (5 activities). As PVF and ALR act as proxy measures of strain magnitude and rate (both of which are important for stimulating the mechanosensory system of bone to result in bone formation [33]), a composite loading score that combined both of these measures (with equal weighting) was created. This score was calculated as the average of the z-scores for peak vertical force and average loading rate (loading rate was log-transformed prior to being z-scored to normalise the positively skewed distribution). The loading score, which is a standardised score representing both the magnitude and rate of strain exerted on the body during activity, can be interpreted as a higher score indicating greater loading. Body mass (kg) was centred (by subtracting the sample mean mass from each subjects’ mass) in order to aid the interpretation of a possible ‘body mass x acceleration’ interaction (i.e., in the presence of the interaction, the acceleration coefficient represents the steepness of the slope when the mass value = 0, which is more meaningful when 0 = mean mass rather than 0 = no mass as no child is 0 kg). This ‘body mass x acceleration’ interaction will determine whether body mass has a modifying effect on the association between acceleration and loading score (i.e., for a given value of acceleration does it predict the same loading score across all participants’ body mass, or does it predict a higher or lower loading score in heavier children?). Maturity offset was dichotomised into pre-PHV (all those with a negative maturity offset value) and post-PHV (all those with a positive maturity offset value) as described by Mirwald et al. [32] to represent distinct stages in biological maturation. As children in study wave 1 and wave 2 had force data collected using different force plates and sampling frequencies (due to limited availability of equipment), a dummy variable was added to the model to determine whether the force plate used had any influence on the outcome by indicating whether there were any systematic differences in loading score between groups.

Linear Mixed-Effects Modelling (LMEM) was used to analyse the repeated measures nature of this data. All participants had 5 repeat measures of acceleration and loading score (one for each type of activity). Therefore, acceleration could be treated as both a fixed effect and a random effect while all other potential predictor variables—including quadratic and cubic terms for acceleration, age, sex, height, centred body mass, maturity status and all two way interactions with acceleration—were entered as fixed effects simultaneously into the model. Entering acceleration as a random effect allowed the acceleration related slopes to vary for each participant. Intercepts were not given the freedom to vary for each participant in these models as the lines should all start in the same place (PVF and ALR = 0 when acceleration = 0). A blended approach of forced entry and manual backwards elimination was used to develop the optimum model for predicting loading score. For the first iteration of the model, all predictor variables listed above were entered simultaneously into the model, and any that were non-significant (*p* > 0.05) in the full model were removed for the second/final iteration of the model unless they were also part of a significant interaction term, or the same variable was significant in the other two accelerometer models. The Pseudo R^2^ and Pseudo R^2^ change were used to identify the proportion of the variance explained by the model and any significant additional variance explained by other factors that were added to the model. The residual plots from the final model were inspected to ensure that residuals were normally distributed and unrelated to the magnitude of the predicted value. Significance was set at ≤0.05. All statistical analyses were conducted using IBM SPSS version 28.0 (IBM, Armonk, NY, USA).

## 3. Results

### 3.1. Participant Characteristics

Descriptive characteristics are presented in Table 1. Due to a technical error with the force plate during wave 1 data collection, 13 participants did not have any ground reaction force data and were excluded from the analysis, resulting in a final sample size of 269 participants (127 boys, 142 girls). Participants had a mean age of 12.3 (±2.0) years, and boys and girls in the sample were similar in terms of age, height, leg length and mass. Girls had a significantly higher BMI compared to boys (19.45 vs. 18.43 kg/m^2^, *p* < 0.05) and a significantly lower predicted age of PHV (12.02 vs. 13.60 years, *p* < 0.05). Maturity offset (years to/from PHV) was also significantly lower in girls than in boys (0.31 vs. −1.28 years, *p* < 0.05).

### 3.2. Linear Mixed-Effects Modelling

**Figure 1:**
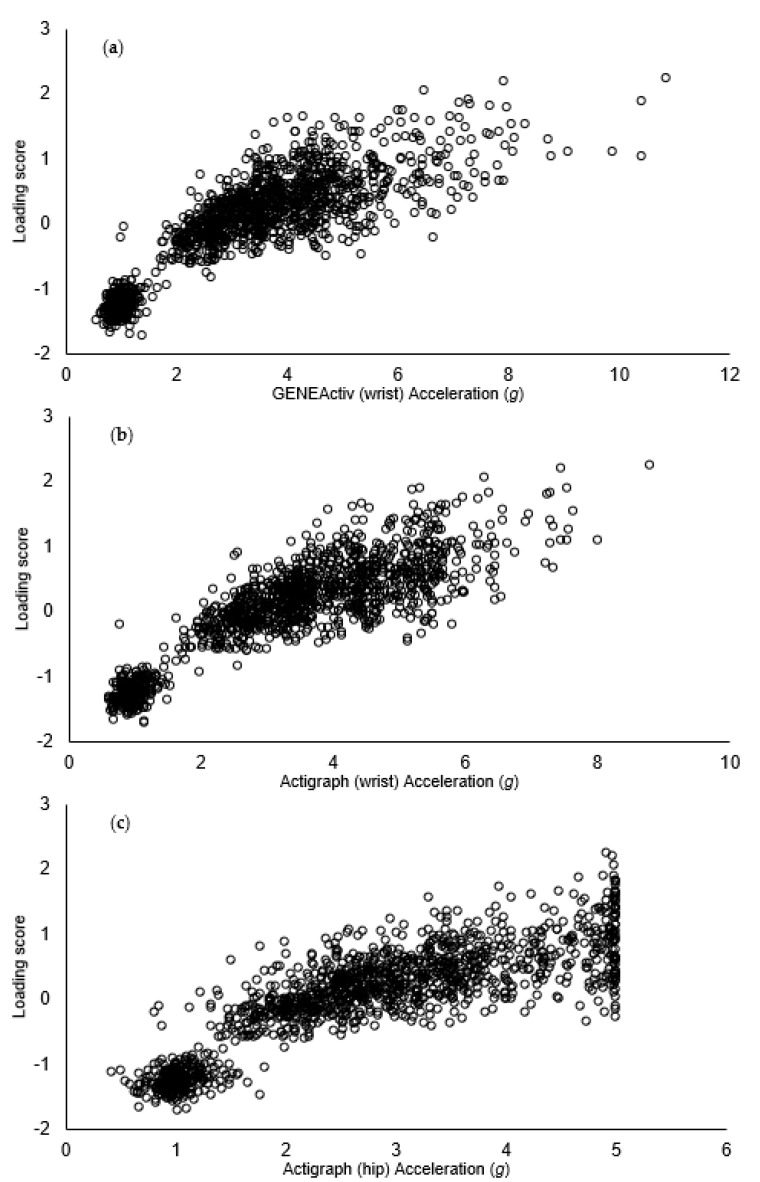
Force loading score and raw acceleration from GENEActiv (**a**), Actigraph wrist (**b**) and Actigraph hip (**c**) accelerometers for all activities for the whole sample. Force loading score was calculated as the average of the z-scores for peak vertical force and average loading rate (loading rate was log-transformed prior to being z-scored to normalise the positively skewed distribution). The loading score can be interpreted as a higher score indicating greater loading. Raw acceleration is presented as the Euclidean norm minus 1 (ENMO).

Scatterplots for the loading score and raw acceleration values from each accelerometer for the whole sample are displayed in Figure 1. Residuals were normally distributed and inspection of residual plots demonstrated that the variance of the residuals did not differ by the magnitude of the predicted value. Sex, age, height, their respective interactions with acceleration, and the ‘maturity x acceleration’ interaction were excluded from the final model as they were all non-significant in the first iteration of the model. In the final model, acceleration was a significant predictor of loading score—accounting for 74.4%, 77.4% and 75.1% of the variance in loading score for the GENEActiv wrist, Actigraph wrist and Actigraph hip, respectively (*p* < 0.001). Given that this relationship was curvilinear, the addition of the quadratic and cubic acceleration terms significantly improved the ability of acceleration to model the loading score (pseudo R2 increased to 80.7%, 81.5% and 79.4% with the addition of these terms for the GENEActiv wrist, Actigraph wrist and Actigraph hip accelerometer, respectively; all *p*’s < 0.001). The unstandardized beta coefficients, 95% confidence intervals, t statistics and *p* values from the final model for the GENEActiv wrist, Actigraph wrist and Actigraph hip are presented in Table 2.

Maturity status (Pre/Post PHV) was a significant predictor of loading score in all models. At the wrist, being pre-PHV was associated with a lower loading score—reflected by the negative coefficients for the pre-PHV group (GENEActiv wrist: unstd beta = −0.14, *p* < 0.001. Actigraph wrist: unstd beta = −0.13, *p* < 0.001; Table 2) whereas at the hip, being pre-PHV was associated with a higher loading score (Actigraph hip: unstd beta = +0.07, *p* = 0.049). Body mass was a significant predictor of loading score in the GENEActiv model (*p* = 0.041). However, for the Actigraph wrist and hip, a significant ‘mass × acceleration’ interaction was observed (*p* = 0.033 and *p* = 0.007, respectively), whereby for a given acceleration value, those who are heavier will produce a slightly lower loading score, and those who are lighter will produce a slightly higher loading score. The final models explained 81.1% (GENEActiv wrist), 81.9% (Actigraph wrist) and 79.9% (Actigraph hip) of the variance in loading score (all *p*’s < 0.001). Entering acceleration as a random effect and allowing the acceleration related slopes to vary for each individual also significantly improved the final model (GENEActiv wrist: unstd beta = 0.002, Wald Z = 6.12; *p* < 0.001; Actigraph wrist: unstd beta = 0.002; Wald Z = 5.89, *p* < 0.001; Actigraph hip: unstd beta = 0.005, Wald Z = 7.47, *p* < 0.001). No significant differences in loading score were identified between children in wave 1 (force data collected using the AccuSwayPLUS force platform) and wave 2 (force data collected using the Accupower force platform; *p* = 0.22–0.87).

## 4. Discussion

The present study examined the associations between peak magnitudes of raw acceleration from wrist- and hip-worn accelerometers and GRF across a range of impact intensities in a large sample of children and adolescents to determine whether raw acceleration can be used as a proxy measure of loading in this population. Using linear mixed-effects modelling to account for the repeated nature of the data, this study found that raw acceleration from both wrist- (GENEActiv, Actigraph GT3X+) and hip-worn (Actigraph GT3X+) accelerometers was strongly and significantly associated with loading (a composite loading score; the mean of PVF and ALR z-scores) in children and adolescents. Body mass and maturity status (pre/post-PHV) were also significantly associated with loading, whereas age, sex and height were not identified as significant predictors. Raw acceleration alone explained ~75% of the variance in loading score. Findings demonstrated that the relationship was curvilinear. Therefore, inclusion of cubic and quadratic acceleration terms further improved the ability of the model to predict loading. The final models for the GENEActiv wrist, Actigraph wrist and Actigraph hip accelerometers explained 81.1%, 81.9% and 79.9% of the variation in loading score, respectively. Results from the present study provide further evidence that accelerometers that output raw acceleration are appropriate for use to monitor the loading exerted on the skeleton. This study also expands the existing literature in child/adolescent populations and demonstrates that wrist-worn accelerometers are also suitable to assess loading in this population.

Whilst studies investigating the associations between raw acceleration and GRF variables in children and adolescents are scarce, similar findings to the present study have been observed. Meyer et al. [19] reported correlations of 0.89 and 0.90 between raw acceleration from hip-worn accelerometers (GENEActiv; Actigraph GT3X+) and peak vertical GRF in 5–16-year-olds for walking, jogging, running, jump landings, skipping and dancing activities. Pouliot-Laforte et al. [14] also reported correlations of 0.96–0.99 between raw acceleration from the hip and GRF during multiple one- and two-legged jumping activities, during a heel rise test in a sample of healthy controls (*n* = 14; aged 6–21 years) and in those with Osteogenesis Imperfecta (*n* = 14; aged 7–21 years). Whilst the latter study did not investigate whether any other factors influenced the relationships observed, in the former study, sex, age, height, weight and leg length were not found to have a significant influence. However, this study used a small sample (*n* = 13) that had a wide age range (5–16 years) and consisted of only three girls. Therefore, it was likely that there was not adequate statistical power to identify any differences due to these factors. In agreement with the present study, others [7,9] have reported that body mass is a significant predictor of force when investigating the associations between acceleration (count-based outputs, 15 and 60 s epochs) and GRF variables. In addition to body mass, the present study also found that maturity status (pre/post-PHV) was significantly associated with loading score. To our knowledge, this is the first study to have investigated whether there is an influence of maturity on the associations observed between acceleration and force.

Maturity status (pre/post-PHV) was a significant predictor of loading score in all models. However, the direction of the relationship differed between the wrist and hip wear locations. At the hip, it was demonstrated that being post-PHV was associated with a slightly lower loading score than pre-PHV. During activities such as jumping, landing peak vertical GRF and loading rate have been shown to decrease from pre- to post-maturity [27,28,29,37]. This is due to improved dampening mechanisms (greater pre-activation and engagement of the stretch-reflex), which serve to effectively reduce stiffness upon landing and the spikes in GRF during ground contact [27,29,37]. Since acceleration measured at the hip provides a good approximation of the forces acting on the body [8], the finding that those in the post-PHV group will have a slightly lower force compared to those who are pre-PHV for the acceleration model at the hip is therefore in agreement with the findings outlined above. At the wrist, being post-PHV was associated with a greater loading score. This opposite relationship may be a result of greater arm movement/noise in the acceleration data at the wrist in less mature children that is unrelated to the loading experienced during impact activities. During gait, less mature children have a more unstable coordination pattern with arm movement, and greater variability in arm swing patterns is observed until 10–14 years of age [38,39]. After this point, arm movement is much more consistent and reflects that of adults [38,39]. Poorer coordination and greater within-subject variability in arm movement patterns may therefore result in higher acceleration values being recorded at the wrist in pre-PHV children compared to post-PHV for a given force and may be why these findings between maturity status and acceleration were identified at the wrist.

Previous studies investigating the associations between raw acceleration and GRF in children and adolescents [14,19] have only used monitors placed on the hip. The present study further expands the literature by investigating associations between loading and raw acceleration from wrist-worn devices. The hip is a popular accelerometer wear location as is it thought to best reflect whole body movement and energy expenditure [40]. It is also an appropriate wear location when assessing activity in relation to loading as the accelerometer measures the second derivative of the displacement of the hip, which is closely related to weight-bearing movement [9]. However, wear compliance with hip-worn devices can be poor [20,25], which leads to selection bias and misclassification [20,40]. Due to the significantly greater wear-compliance observed with wrist-worn monitors [20,24,25], they are now increasingly used to assess PA in relation to numerous health outcomes. Results in the present study demonstrated that the relationship between raw acceleration and loading was very similar for both the wrist- and hip-worn accelerometers, which suggests that wrist-worn devices that output raw data are also appropriate for use when assessing PA in relation to loading in children and adolescents. Comparable relationships between wrist- and hip-worn accelerometers have also been reported in similar studies in adults [15,16]. However, further research is needed to confirm whether these findings translate to free-living situations where activities that involve decoupling of wrist and hip accelerations occur [25,40,41].

The use of raw acceleration data, rather than data processed in any epoch length, is a strength of the present study as it prevents over-smoothing of brief, sporadic bursts of high-intensity activity that are important for generating an osteogenic response [34]. The multilevel regression analysis used also offers merit over other analysis approaches, such as performing correlations for each activity. At present, it is not possible to know the type of activity being performed in free-living data, so although correlation analysis per activity is useful, it does not demonstrate how well loading can be predicted without information on activity type. Using the multilevel regression approach, findings demonstrate that you can explain ~80% of the variance in loading using accelerometry, even when there is no information regarding activity type. The use of a large sample is also beneficial as it enables the influence of factors including age, sex, height, weight and maturity status on the associations between raw acceleration and loading to be investigated in more detail. Nevertheless, this study is not without limitations.

The use of a portable force plate rather than a laboratory mounted force plate may be a limiting factor. Whilst laboratory mounted force plates are considered the ‘gold standard’, a number of studies have demonstrated that portable force plates are reliable, precise and accurate and provide data that are highly comparable to those of laboratory-based plates [42,43,44,45]. The GRF data were also collected using two different force plate models with different sampling frequencies (200 Hz vs. 1000 Hz, due to technical specification). Whilst there is a risk that the lower resolution of 200 Hz may result in missed impact peaks [45], studies have reported near perfect correlations between peak force data collected at 200 Hz during jumping and data sampled at 250, 400 and 500 Hz [46]. Moreover, data sampled at 400 Hz have been shown to capture very similar data to those of 1200 Hz [45]. Therefore, the different sampling frequencies are unlikely to have impacted the force data obtained. There was also no evidence of systematic differences between the measures in the regression model. Whilst similar associations between raw acceleration and loading were identified for the wrist and hip wear-locations in this sample, the study included structured activities where arm movements typically paralleled lower body movements. The similar findings between wrist and hip wear locations may, therefore, not translate to free-living situations where decoupling of wrist and hip accelerations have been shown to occur, and accelerations during particular activities are disproportionately larger at one location compared to the other [25,40,41]. For example, during racket sports, basketball and computer games, the wrist will record disproportionately larger accelerations than the hip [40]. The decoupling of wrist and hip accelerations, and therefore the extent to which the relationship between loading and acceleration is similar, will be population specific [40], and further research is needed to investigate whether these findings translate to free-living situations with more unstructured activities, or whether differences between the wrist and hip wear locations occur. Free-living, unstructured situations are also likely to result in more noise in the acceleration signal, and the use of a filter may therefore need to be explored and applied in the future in order to get a clean estimation of body accelerations. It should also be noted that reliability and validity of the Mirwald method for estimating age at PHV in youth has been questioned, and thus, the findings of this study pertaining to the influence of maturation should be interpreted with a degree of caution [47]. Future studies may seek to replicate this study with alternative methods for estimating biological maturation. Finally, the Actigraph accelerometer reached its measurement limit for some of the high-impact jumping activities. In future, devices with a larger measurement range may be needed to detect all movements that incur large mechanical loads.

The present study demonstrates that raw acceleration from both wrist- and hip-worn monitors is a valid approach to measuring the loading incurred during PA in children and adolescents. The finding that the wrist performed similarly to the hip is particularly encouraging as adherence to wear protocols is much higher with wrist-worn monitors, and the activity data obtained are therefore more representative of habitual activity [20,40]. As raw acceleration is reflective of the loading incurred during PA and has the resolution necessary to examine impact peaks in the data over several days/weeks, future research should examine the frequency related mechanisms of loading in relation to bone health outcomes. The ability to accurately capture exposure to loading over longer periods of time in free-living situations using accelerometers and determining the temporal aspects of dynamic loading will enable new insights to be gained as to how habitual PA influences skeletal health during growth.

## 5. Conclusions

The present study examined the associations between ground reaction force variables and peak magnitudes of raw acceleration from both hip- and wrist-worn accelerometers in a large sample of children and adolescents to determine whether raw acceleration is a valid approach to measuring the loading incurred during PA in this population. Linear mixed-effect regression modelling demonstrated that raw acceleration from both hip- and wrist-worn accelerometers is strongly and significantly associated with loading in children and adolescents (explaining around 80% of the variance) and is therefore a suitable method of assessing the loading characteristics of PA in the future.

## Figures and Tables

**Table 1:** Descriptive characteristics for the whole study sample, and for boys and girls separately. Mean (SD).

	All	Male	Female
	(*n* = 269)	(*n* = 127)	(*n* = 142)
Age	12.3 (2.0)	12.3 (2.1)	12.3 (1.9)
Height (m)	1.54 (0.14)	1.55 (0.15)	1.54 (0.12)
Leg Length (m)	0.74 (0.07)	0.75 (0.08)	0.73 (0.07)
Mass (kg)	46.01 (13.50)	45.15 (13.75)	46.79 (13.27)
BMI (kg/m^2^)	18.97 (3.12)	18.43 (2.87)	19.45 (3.27) *
Predicted APHV	12.77 (1.02)	13.60 (0.69)	12.02 (0.60) *
Maturity offset (years)	−0.44 (1.93)	−1.28 (1.86)	0.31 (1.68) *
Pre/Post-PHV (%)	53/47	68/32	40/60

BMI = body mass index; APHV = age at peak height velocity; prePHV = those with a negative maturity offset value; postPHV = those with a positive maturity offset value; maturity offset predicted using Mirwald et al. [32] prediction equations; * *p* < 0.05 for differences between boys and girls.

**Table 2:** Relationship between loading scores and raw acceleration values from the GENEActiv wrist, Actigraph wrist and Actigraph hip accelerometers (statistics from linear mixed-effects regression models).

	GENEActiv (Wrist)			Actigraph (Wrist)			Actigraph (Hip)		
	Unstd Beta Coeff (95% CI)	*t*	*p*	Unstd Beta Coeff (95% CI)	*t*	*p*	Unstd Beta Coeff (95% CI)	*t*	*p*
Intercept	−2.12 (−2.29, −2.10)	−45.61	<0.001	−2.25 (−2.35, −2.14)	−40.72	<0.001	−2.98 (3.17, −2.80)	31.67	<0.001
Acceleration (g)	1.21 (1.12, 1.29)	28.39	<0.001	1.30 (1.19, 1.42)	22.53	<0.001	2.22 (1.97, 2.46)	17.69	<0.001
Acceleration squared (g)	−0.17 (−0.19, −0.15)	−15.34	<0.001	−0.21 (−0.25, −0.18)	−12.25	<0.001	−0.52 (−0.061, −0.42)	−10.56	<0.001
Acceleration cubed (g)	0.01 (0.01, 0.01)	10.87	<0.001	0.01 (0.01, 0.02)	9.18	<0.001	0.05 (0.04, 0.06)	8.25	<0.001
Maturity status (prePHV = 0, postPHV = 1)	−0.14 (−0.2, −0.07)	−4.24	<0.001	−0.13 (−0.19, −0.07)	−4.03	<0.001	0.07 (0.00, 0.14)	1.97	0.049
Body mass ^c^ (kg)	−0.003 (−0.01, 0.00)	−2.04	0.041	−0.002 (−0.01, 0.001)	−1.21	0.229	0.003 (0.00, 0.01)	1.74	0.082
Body mass ^c^ × Acceleration interaction	0.0005 (−0.001, 0.0004)	−1.01	0.312	−0.001 (−0.002, −0.0001)	−2.13	0.033	−0.002 (−0.003, 0.0005)	−2.71	0.007
Goodness of fit	Pseudo R^2^ (%) AIC	81.1 887.1			81.9 839.7			79.9 896.4	

Unstd = unstandardized; AIC= Akaike’s information criterion; CI = confidence interval; PHV = peak height velocity (all negative maturity offset values categorised as prePHV and all positive maturity values categorised as postPHV, estimated by Mirwald et al. [32] prediction equations); g = gravitational units (where 1 g = 9.81 m.s^2^); ^c^ = centred on the mean body mass of the whole sample.

## Data Availability

The data presented in this study are available on request from the corresponding author.
